# Traditional Self-Reported Dietary Instruments Are Prone to Inaccuracies and New Approaches Are Needed

**DOI:** 10.3389/fnut.2020.00090

**Published:** 2020-07-03

**Authors:** Michele N. Ravelli, Dale A. Schoeller

**Affiliations:** ^1^Department of Neurology, University of Wisconsin, Madison, WI, United States; ^2^Nutritional Sciences and Biotechnology Center, University of Wisconsin, Madison, WI, United States

**Keywords:** diet records, self-report, energy intake, underreporting, doubly labeled water method

## Abstract

**Background:** Diet is a modifiable behavior that influences an individual's health. Because of this, diet assessment is an important component of public health surveillance, evaluating response to community health interventions, and monitoring individual compliance to medical interventions. Diet assessments are usually performed using one of three basic methods: diet recall, diet diaries, or food frequency questionnaires. Although these three assessment instruments have displayed a strong agreement between themselves, when reported intake is compared with intake measured using quantitative nutrient biomarkers, investigators have identified systematic misreporting errors for all three of these self-reported dietary instruments.

**Aims:** This work aims to summarize the state of knowledge regarding misreporting and why it impedes diet–health research and to introduce advances in the collection and the treatment of dietary data.

**Methods:** This work reviews and summarizes published data on misreporting and the recent efforts to reduce such errors.

**Results:** The evidence demonstrates a strong and consistent systematic underreporting of energy intake (EIn) across adults and children studies. Underreporting of EIn has been found to increase with body mass index (BMI), and the differences between macronutrient reports indicate that not all foods are underreported equally. Protein is least underreported, but which specific foods are commonly underreported are not known.

**Conclusions:** Because energy underreporting varies as a function of BMI, self-reported EIn should not be used for the study of energy balance in the study of obesity. The between-individual variability in the underreporting of self-reported intake of energy and other nutrients attenuates diet–disease relationships. Recent efforts to correct for underreporting have reduced misreporting of diet outcomes, but improvements have been incremental in nature and more research is needed to validate and extend these efforts.

## Introduction

Investigations into the role of diet in the development of disease in humans are viewed as difficult but important because diet is one of the behaviors that individuals may employ to maintain or improve health (1). For example, human feeding studies performed or dictated by external disruptions of food supplies have clearly demonstrated that diet plays a central role in the negative impacts on health resulting from energy, vitamin, and mineral deficiencies in controlled studies conducted for at least 75 years (2). At the same time, the Minnesota Semi-starvation Study demonstrated the difficulty and the ethical issues of performing month-long feeding trials in order to investigate the quantitative nature of diet–disease relationships using only an inpatient paradigm (3).

A major source of difficulty has been documenting what individuals consume as their typical diet due to dietary measurement error (4). Because controlled and thus more accurate feeding studies are costly and difficult to perform, a far more common approach to the study of the relationship between diet and disease has been to perform studies of free-living participants. These studies usually rely on assessing diet using self-report instruments to access diet and this introduces diet measurement error into the study (5). The most common dietary assessment instruments are diet recall surveys, in which the participants report from memory each item of food consumed for the previous day, weeks, or months; food frequency questionnaires (FFQ), which have the aim of assessing the food consumption during some specified period of time or over a period such as adolescence; and diet diary methods where the subjects record dietary intake for each eating event for a period of days or weeks.

Perhaps the best documented evidence of dietary instrument measurement error is that of self-reported energy intake (EIn) being often less than that of the individual's measured energy expenditure. The aim of this short review is to summarize the development of that evidence and how misreporting impedes diet—health research. Because of the evidence, there has been a renewal of efforts to improve or develop alternatives to these instruments. These recent advances are introduced here as part of a series of reviews in this issue.

## Dietary Misreporting

Over the past 50 years, many traditional diet assessment instruments have undergone modification of content or structure, sometimes to the degree of being considered a new instrument, and then were validated by comparison against an older version or previously evaluated instrument. In most instances, these comparisons demonstrated a moderate or strong agreement between the basic types of dietary assessment instruments and the tool was considered to be of reasonable accuracy and precision (6). A few investigators, however, performed studies comparing self-reported dietary intake against a biomarker of dietary intake, such as urinary nitrogen, which provides an objective measure of dietary protein intake (7). Such comparisons against a biomarker often did not find these self-report instruments to be accurate. For example, Warnold et al. (8) reported that self-reported protein intake underestimated protein consumption by 47% compared to protein intake measured using urinary nitrogen outputs among women undergoing a weight loss treatment. Studies comparing self-report against biomarkers to test the accuracy of traditional diet instruments, however, were infrequent and had only a modest influence on the growth of the use of self-report dietary assessments in the study of diet–disease relationships.

The number of dietary instrument validations against a biomarker increased dramatically following the development of the doubly labeled water (DLW) method to measure total energy expenditure (TEE) in humans (9). This method, developed by Lifson, is based on the difference in the elimination kinetics of two stable isotopes in water, namely, deuterium (^2^H) and ^18^O (10). The difference in the elimination rate of ^2^H and ^18^O is proportional to carbon dioxide production (10). The latter is the end-product of oxidative phosphorylation, and TEE can be calculated using standard indirect calorimetric equations (11). The human validations that included conditions of weight stability, overfeeding, underfeeding, intravenous feeding, and heavy exercise have been summarized by Speakman et al. (12–15). These validations have displayed an average accuracy of TEE of 1 to 2% and an individual precision of 7%, which support its use as a biomarker for use in a criterion method against which one may test the accuracy and the precision of self-reported energy intake.

The development of the DLW method for the measurement of TEE created an opportunity to validate diet assessment instruments against an objective energy expenditure based on the first law of thermodynamics. The first law of thermodynamics states that energy cannot be created nor destroyed, and thus EIn equals energy expenditure plus or minus the change in body energy stores during the measurement interval. Moreover, when body energy stores are unchanged over time, the energy storage term falls to zero and then EIn equals energy expenditure. Among pregnant women, infants, and children, change in energy stores over time is expected. At 1 month of age, the average daily energy storage is about 40% of EIn, but this decreases to 3% by 1 year of age and continues to decrease to 1% at 10 years of age (16). Among pregnant women, the average increase in body energy stores is about 190 kcal/day or 8% of EIn during the 3rd trimester (17). Among most non-pregnant adults, weight gain is not recommended, but it is common. The rate of weight gain is 0.2–0.7 kg/year or about 1–2 g/day (18). Assuming that the gain is adipose tissue with 20% fat-free mass and 80% fat mass, this stores only about 8 to 16 kcal/day or about 0.3–0.6% of EIn (19) and thus energy expenditure almost equals EIn, and TEE is an excellent biomarker of EIn. There are exceptions to this essential near-equality assumption in adulthood. These include periods of voluntary weight loss, loss of appetite during illness, or periods of holiday feasts when energy intake can be quite different from expenditure and TEE will fail to be a quantitative biomarker of actual EIn. Under habitual conditions outside of these short periods, EIn roughly equals TEE. Thus, TEE is termed as a biomarker of habitual dietary energy intake rather than one of actual EIn. This is an important distinction because while TEE is generally a good measure of habitual energy intake for the study of diet and health, it fails as a measure of actual energy intake under the above mentioned short-term conditions.

Another consideration in using TEE as a biomarker of habitual EIn is that energy intake can be expressed in one of three ways. The first is gross energy. This is the total energy available when foods are combusted to dioxide carbon, water, and nitrogen gas using bomb calorimetry (20). Not all the gross energy, however, is available to the body for metabolism. About 8% of the gross energy is not absorbed and thus lost from the body as waste products in feces (21, 22). The second expression is the absorbed energy. Finally, not all the absorbed energy is available to the body for energy production because some compounds that still contain chemical energy are lost as waste products in urine. The third expression is the portion of absorbed energy retained by the body that is available for energy production, which is the metabolizable energy. Metabolizable energy, as the name implies, is available for use in oxidative phosphorylation. This is the energy value listed in the food handbooks and tables. Metabolizable energy is thus the energy value used for calculating the dietary EIn by dietary assessment instruments.

Many of the human studies using DLW performed during the 10 years between 1982 and 1992 included an assessment of dietary EIn using traditional instruments. One of the first studies was conducted by Prentice et al. (23), in which it was observed that EIn, assessed using a 7-days food diary, was 34% (*P* < 0.05) less than TEE measured by DLW in young adult obese women (32.9 ± 4.6 kg/m^2^), but there was no difference detected between EIn and TEE (2%, NS) in lean women. The authors also found that half of the EIn vs. TEE difference was due to underrating as assessed by weight loss during the dietary diary period. These findings of low self-reported EIn were confirmed in a later review (24), which included papers which found that underreporting of dietary EIn was observed in women with anorexia nervosa, who perceive that they have excess body fat, and also in individuals with measured excess body weight, who are concerned about actual excess fat. Thus, underreporting was associated with individuals likely to be concerned about excess weight and not just with actual weight status (body mass index, BMI) itself (24). Thus, as early as 1990, it was found that underreporting of dietary EIn was common among adults and linked to concerns regarding excess body weight or fat (24). Even these early studies found that the degree of underreporting was of similar magnitude regardless of whether intake was assessed using retrospective instruments such as diet recalls or histories or with instruments such as food diaries (24). Based on these observations, it was concluded that dietary assessment instruments were subject to errors that increased with the individual's concern regarding their relative weight, which would result in a correlation of increased underreporting with increased BMI (24). Because of this, it was strongly recommended that self-reported EIn should not be used as a primary assessment instrument to measure EIn in investigations into the role of EIn in weight regulation as early as 1990 (24).

Most of these early studies employing DLW as a quantitative biomarker of dietary EIn were conducted in cohorts with sample sizes categorized as small to medium and, in many cases, by investigators without extensive experience on the use of dietary assessment instruments. Based on anecdotal evidence provided by questions from the audience following oral presentations, some investigators in the audience suggested that the finding of underreporting may have been an artifact and that it might not occur if experienced investigators performed studies in large cohorts. This hypothesis, however, was not supported by the results from one study and soon thereafter by four more studies that were performed by investigators who had extensive experience on the use of dietary assessment instruments and which included cohorts with several hundreds of adult subjects each as summarized by Freedman et al. (25). The combined results of these studies confirmed that underreporting of habitual EIn in the United States was common as it was observed in each of the five studies which, when combined, involved over 2,000 participants (25). The 24-h recall (24HR) exhibited an EIn underreporting compared to the DLW-measured TEE which averaged −16% (range, −10 to −28%), and the FFQ was subject to an even larger reporting error than was 24HR (range, −26 to −32%) (25). The combined number of participants in these five studies (*n* = 2,265) permitted sub-analyses, and it was found that those having a BMI of >30 kg/m^2^ underreported EIn by 7% more than those of a BMI in the healthy range, but there was no difference between men and women or adult age groups when centered on ages 50–59 years (25). One of these five studies (26) found that the administration of up to eight 24HRs on different days of the week did not eliminate the average reporting error, thus demonstrating that the underreporting was not simply due to day-to-day variation in actual EIn. The underreporting did decrease when two 24HRs were averaged (−11%) relative to that when only one 24HR was employed (−15%), but the percent error changed only a little when more than two 24HRs were averaged. Even when six 24HR data were collected and averaged, the bias dropped to only −9%. Thus, dietary data were more consistent when two recalls were employed in each participant, but little was gained by further replication.

The findings from the combination of the five large studies discussed above have been confirmed and extended through a systematic review conducted by Burrows et al. (27). The review identified an additional 59 studies that included 6,298 adults, including the abovementioned five studies and the 2,265 participants in the abovementioned summary by Freedman (25). The studies employed a mixture of diet instruments, including 24HR; the food diaries include weighed food records and FFQs. The degree of underreporting relative to habitual EIn as measured by DLW varied over a wide range. This included two studies that reported group averages displaying over-reporting (7 and 8%), but the vast majority identified cohort average underreporting by between 1 and 38%, and the plurality of studies found an average under-reporting between 20 and 30%. A comparison of methods indicated that the most misreporting was observed for the FFQ and the least for 24HR, but all three methods displayed underreporting errors. Studies that included advanced technology such as photography, hand-held personal digital assistants, or oral recordings did reduce the underreporting slightly compared to non-technology-assisted instruments but were still found to be subject to underreporting. Included in that review were studies conducted in countries other than the United States, including Australia, Brazil, Canada, Finland, Germany, Japan, New Zeeland, Norway, Sweden, and the United Kingdom, demonstrating that underreporting with regard to EIn was a global problem.

Two systematic reviews concluded that underreporting of EIn was also an issue among children (28, 29). There have not been as many studies performed in children (aged 3–18 years) as have been performed in adults, but the results were similar with those in adults. Underreporting was common in children, and children with excess weight (overweight or obese) underreported more than those having a BMI in the healthy weight range. It was found that underreporting was reduced when parents assisted their children for ages <11 years. Unlike what was observed in adults, age was a significant modifier of misreporting, and underreporting was greater in adolescents than it was in younger children.

Because of the significant underreporting of EIn observed in the studies discussed above, one of the vital next steps for research directed at studying the phenomenon of EIn underreporting is to identify whether underreporting of EIn is due to a failure to accurately report specific foods or is a general underreporting of all foods. Addressing this issue is difficult because it means one has to measure something that is not reported rather than what is reported. We speculate that one means of accomplishing this would be to include multiple biomarkers in a study of self-reported dietary intake. As evidence, studies that have included DLW as a biomarker for EIn and urinary nitrogen as a biomarker for protein intake have shown that energy is underreported by a larger percentage than protein. For example, the abovementioned study combining the results from five large dietary intake studies (25) found that while energy was misreported by −16% (range, −6 to −28% using 24HR), protein was misreported by only −5% (range, −21 to +20%), indicating that protein was not as underreported as carbohydrate and/or fat. We speculate that a cluster analysis using multiple quantitative and possibly semi-quantitative biomarkers will provide vital insight into the foods that are misreported. The value of identifying what foods were being underreported as well as the difficulty of doing so without using biomarkers is illustrated by a Brazilian study performed in obese women prior to bariatric surgery (30). The study found that the under-reporters reported lower intakes of foods with high energy density but with similar intakes of calories provided by healthy foods (fruits, leafy vegetables, and vegetables) compared to those of plausible reporters. This reporting behavior influenced the determination of dietary patterns by exploratory factor analysis, in which the principal component analysis with VARIMAX rotation was applied for the selection of food groups that composed the matrix and then used for dietary pattern interpretation (30). By combining diet factor analysis with biomarker data on energy, protein, sugar, sodium, and potassium, it should be possible to infer if these differences were due to actual dietary intake differences.

Misreporting of energy and protein intake when assessing diet by self-report is well-documented and recognized by many as a major limitation to the investigation of the effects of diet on health. The problem of underreporting, particularly because of the inter-individual variation in misreporting, dramatically attenuates diet–disease relationships. Kipnis et al. (31) modeled the effects of misreporting of protein and EIn in the OPEN study and concluded that the variation in the degree of misreporting using an FFQ would severely attenuate the relative risk between true protein or EIn and disease from a true value of 2.0 to an apparent relative risk of <1.1. Even worse, it may even reverse the association between diet and disease as had occurred in an analysis of energy balance using self-reported EIn and physical activity by Kromhout et al. (32). The data from these investigators indicated that energy balance and BMI were negative and becoming more negative with increasing BMI, a result that they considered implausible and a possible artifact of underreporting EIn.

In summary, the problem of misreporting of dietary intake is limiting the ability of investigators to study diet–disease relationships (31). Investigators are, therefore, performing studies of novel approaches that may either reduce misreporting or adjust the self-reported data using *post hoc* techniques that may reduce the effect of such misreporting on study outcomes (33). These include the development of advanced technology to reduce the reporting errors themselves, adjustment of reported nutrient intake using calibration against a nutrient biomarker, statistical approaches that provide novel analyses of data from traditional self-reported dietary instruments, or direct use of dietary biomarkers to assess intake (34).

## Approaches to Reduce Misreporting

Advanced technological tools include digital photography with on-line submission, movement monitors on the wrist or eating utensils to detect feeding, microphones to detect chewing, and scales to monitor the disappearance of food from a plate. Photographic methods provide the most detailed information about foods consumed, but they are still prone to underreporting (27) and they require a large amount of technical support (35). The other methods listed above have demonstrated the ability to detect eating events, but they provide only partial quantitative and qualitative information regarding the foods being consumed (34).

In addition, *post hoc* approaches that reduced the influence of misreporting have been presented. For example, Mozaffarian et al. (36) analyzed diet data obtained using an FFQ administered at 4-year intervals in a large longitudinal study. They used dietary change scores from the bracketing FFQs in place of raw intake scores from a single FFQ to identify foods that were associated with 4-years changes in body weight. This approach of using diet change and subsequent weight change is difficult to validate for dietary reporting accuracy, but the foods identified in this study as being associated with weight gain or loss were in general agreement with small, shorter intervention studies and thus extended the findings from the short-term interventions to the population level. Additionally, it provided high statistical power, but it did require a study design where the diet was assessed multiple times over a period of years in a large cohort and an outcome that was continuous. A second *post hoc* data analysis approach was employed by Freedman et al. (37). They combined multiple 24HRs with an FFQ in order to use the quantitative information from 24HR along with the larger list of foods consumed from the FFQ against true intake as measured by dietary biomarkers. They reported an improvement in the correlation coefficients between reported and biomarker-measured true dietary intake of energy, protein, potassium, and sodium compared to the use of single 24HR by an average of 0.14. The highest correlation coefficient, however, was 0.64 for potassium in women, and thus the variance explained was <40%.

As an alternative to the above-discussed methods to reduce problems arising from misreporting, Prentice and Huang (38) have proposed and tested the use of a *post hoc* calibration to adjust reported intakes for misreporting identified by the use of a quantitative biomarker in the entire study cohort or a subsample of that cohort. Tasevska et al. (39) applied this approach to an analysis of self-reported sugar intake and the likelihood of developing type II diabetes or cardiovascular disease. They found that correcting reported sugar intake based on the calibration eliminated what appeared to be an implausible inverse relationship, thus avoided a false finding. The resulting positive correlation, however, was small and did not result in a significant increase in the odds ratio for disease development with increasing sugar intake during the 16-years follow-up in the Women's Health Initiative cohort of older women, thus not ending the controversy around sugar consumption and type II diabetes.

The final approach to be discussed in this review is that of Goldberg et al. (40, 41). This approach involved characterizing a self-reported intake as plausible or implausible. During a period of bodyweight stability, the ratio of $\frac{{energy\;intake\;}_{reported}}{resting\;metabolic\;rate}$ should correspond to the ratio of $\frac{total\;energy\;expenditure}{\;resting\;metabolic\;rate}$, which is identified as physical activity level (PAL). Considering the biological variability of the components of the equation, confidence limits (cutoffs) are calculated to classify the probable accuracy of the reported EIn, and its sensibility improves when individual PAL classification is used in the cutoff points (42). A not dissimilar approach is to calculate the ratio of reported EIn to TEE from DLW (24). As an alternative to the DLW method, it may be possible to use a predicted TEE based on weight, height, age, sex and physical activity (18). The optimal method for defining the cutoff for excluding implausible reported intakes is still under debate, but the value is recognized (43).

## Summary and Conclusion

Dietary assessment is central to the study of diet–health relationships. The most common assessment instruments are diet recalls, diet diaries, and food frequency questionnaires, and all are dependent on self-reported data. Self-reported Ein, using all of these instruments, has been shown to yield reproducible intake results. They have also been shown to yield good to strong correlations between foods consumed when compared against one another. Comparisons against quantitative biomarkers of dietary intake, however, have clearly demonstrated that self-report is prone to misreporting errors for EIn and other nutrients and that inter-individual variability in the degree of underreporting attenuates the strength of diet–disease relationships and raises questions regarding what foods are being misreported. Recent research has identified several methods for reducing many of these reporting errors. There remains, however, a need for further research to optimize the accuracy or correct for inaccuracies in self-reported dietary data because of the importance of dietary data in the prevention and the treatment of diet-induced diseases.

## Author Contributions

MR and DS conceptualized the manuscript. MR and DS drafted and edited the manuscript.

## Conflict of Interest

The authors declare that the research was conducted in the absence of any commercial or financial relationships that could be construed as a potential conflict of interest.
